# Beyond Mood and Atmosphere: a Conceptual History of the Term *Stimmung*

**DOI:** 10.1007/s11406-020-00290-7

**Published:** 2020-11-27

**Authors:** Gerhard Thonhauser

**Affiliations:** Department of Philosophy, Technical University of Darmstadt, Karolinenplatz 5, 64289 Darmstadt, Germany

**Keywords:** Mood, Attunement, Tuning, Atmosphere, Aesthetic experience, Empathy

## Abstract

The last few years have seen increasing research interest in moods and atmospheres. While this trend has been accompanied by growing interest in the history of the word *Stimmung* in other disciplines, this has not yet been the case within philosophy. Against this background, this paper offers a conceptual history of the word *Stimmung*, focusing on the period from Kant to Heidegger, as this period is, presumably, less known to researchers working with notions like mood, attunement or atmosphere today. Thus, considering this period might provide conceptual resources not yet considered in current debate. *Stimmung* has the remarkable feature of encompassing the entire semantic field of mood and atmosphere, insofar as both subjects and objects can literally be in *Stimmung*. *Stimmung* might refer to the state or condition of being attuned, which is understood as a dispositional state, as well as the process or act of attuning, which includes self-activating and foreign-determined forms of attuning. The word was first used for the tuning of musical instruments, but was quickly transferred to the fields of aesthetics, psychology, and physiology. This paper will focus on the contrast between the psychological canonization of *Stimmung* as a type of mental state, and the use of *Stimmung* as an untranslatable, irreducible metaphor with unique semantic force allowing for original theorizing.

## Introduction

This paper offers an investigation into the conceptual history of the word *Stimmung*.^1^ This investigation is conducted against the background of increasing research interest in moods and atmospheres.^2^ While in other disciplines this trend has been accompanied by growing interest in the word *Stimmung* and its history,^3^ this has not yet been the case within philosophy.^4^ This can be identified as a lacuna in current debates, which do not pay sufficient attention to the history and semantic content of their core concepts.

*Stimmung* has the remarkable feature of encompassing the entire semantic field of mood and atmosphere, insofar as both subjects and objects can literally be in *Stimmung*.^5^ The German language allows to speak of the ‘*Stimmung* of a meeting’ or the ‘*Stimmung* of a room’ just as natural as of ‘my *Stimmung’*. The word combines “the objective (factual) and the subjective (psychological) into one harmonious unity“ (Spitzer 1963, 5). Thus, considering the emotional and atmospheric use of *Stimmung* has the potential to provide historic depth and conceptual potential to current research on mood and atmosphere.

My research is guided by the thesis that *Stimmung* is an example of what Blumenberg (1997) called an “absolute metaphor.” The emotional and atmospheric use of *Stimmung* is such that it cannot be translated into non-metaphorical terms. On the contrary, what the term *Stimmung* addresses first obtains its meaning from the metaphor of *Stimmung* and the semantic field of musical attunement it calls into play. As we will see throughout this paper regarding various discourses, *Stimmung* as an absolute metaphor is constitutive of the conceptualization of relevant terms as well as the grasp of relevant phenomena and, thus, governs the discourses which it enables.

The following section begins with a brief look into the etymology, possible translation and early history of the word *Stimmung*, providing a first delineation of the core semantic content of the *Stimmung* metaphor (Section 2). Then, I will offer a detailed reconstruction of the conceptual history of the term from Kant to Heidegger, which can be divided into four stages: Kant’s *Critique of Judgment* (1790), which appropriated the *Stimmung* metaphor for the theory of aesthetic judgment, served as a major landmark in the history of the concept (Section 3). In the initial stage following Kant, *Stimmung* was a lively metaphor, displaying its full semantic complexity and allowing for original conceptual work (Section 4). In a process that dates back prior to Kant and was ongoing throughout the nineteenth century, *Stimmung* was transformed into a psychological concept. This process culminated in psychological discourse at the turn of the twentieth century, which canonized *Stimmung* into psychological vocabulary (Section 5). Parallel to this process of psychologizing *Stimmung*, we find alternative approaches that are closer aligned with the original metaphor. Those attempts are centred around the aim of clarifying the *Stimmung* (here best translated as atmosphere) of phenomena like artworks, landscapes or weather conditions (Section 6). Finally, the work of Martin Heidegger can be interpreted as an attempt to explicate the ontological alternative implicated in those alternative approaches to *Stimmung* (Section 7). Of course, much could be said about work on *Stimmung* after Heidegger. However, this paper focuses on the period up until Heidegger, as this period is, presumably, less known to researchers working with notions like mood, attunement or atmosphere today. Thus, considering this period might provide conceptual resources not yet considered in current debate. Nevertheless, some important developments post Heidegger will be sketched in a brief epilogue in which I will also draw some tentative conclusions for current research (Section 8).

## Etymology, Translation and Prehistory

The term *Stimmung* derives from the root *Stimme* (voice), related to the Old Greek *stoma* (mouth). Despite this original reference to the human body and expression, the abstract noun *Stimmung* was first used with reference to musical instruments. It described the tuning (*stimmen*) of instruments, in which case they are tuned (*gestimmt*). The common use of *Stimmung* first started in the eighteenth century. The path from *Stimme* (voice) to *Stimmung* (attunement) was likely via the verb *stimmen* (to attune) and the adjective *stimmig* (harmonious, more verbatim: having the same voice) (Grimm and Grimm 1854; Kluge 2012).

Let us briefly consider two potential English translations. First, the words *tuning* and *attunement* share the musical origin of *Stimmung*. However, they have a rather technical accentuation, which makes their attribution to subjects quite artificial.^6^ Tuning can not only be used with reference to musical instruments, but has a broad range of technical applications, where it usually means the optimization of a system. A commonly known example is car tuning. Similarly, we speak of the tuning of an engine when parameters are adjusted or modified to improve performance. In the natural sciences, model tuning means the calibrating of parameters in a model so the model better fits target data, for instance in climate science (Frisch 2015; Schmidt et al. 2017). In physiology, tuning is used to describes both the attribute of neurons to only respond to specific information and the mechanisms through which neurons reach such selectivity (Sakai et al. 1994). This list could certainly be continued.

Second, the most obvious translation of *Stimmung* is mood.^7^ However, in contrast to tuning and attunement, the term *mood* lacks any reference to music. The English word mood shares the root with the German term *Mut.*^8^ Both originally meant mind, thought or will. Thus, mood, as well as it correlates in German, were from the beginning located in the semantic field of the mind and do not allow for technical applications. Derived from this root is the German term *Gemüt*, which signifies the unity of the powers of the mind and plays a crucial role in eightenth and nineteenth century discourse.^9^

Already in the middle of the eighteenth century, hence shortly after the word was coined, *Stimmung* was transferred from the musical domain to the emerging fields of aesthetics, psychology, and physiology (Welsh 2009a). In medical discourse the notion of *Stimmung* was applied to the physiology of nerves and the brain (Hartley 1749; Weikard 1790). Towards the end of the eighteenth century, phrases like “Stimmung des Gemüts” (attunement of the mind) (Sulzer 1777, 776) and “Stimmung der Lebenskraft” (attunement of vital force) (Reil 1910 [1795], 10–11) were widely spread among different fields of knowledge (Welsh 2009a, 149–52). Kant could build on this established use of the term when writing his *Critique of Judgment* in 1790.

But before I proceed with discussing Kant’s text, let me briefly summarize the core semantic content of the *Stimmung* metaphor (Welsh 2009a, 48; Wellbery 2003, 707–9): First, the metaphor of *Stimmung* allows to focus on the state of being attuned (of a musical instrument, the mind, or the nervous system). It alludes to a state of harmonious attunement, but it might also imply a change of attunement (*Umstimmung*) or a detuning (*Verstimmung*). Second, the state of being attuned is understood as a dispositional state. Similar to the state in which an instrument is ready to be played, an attuned mind (*gestimmtes Gemüt*) is ready to be determined (*bestimmt*). The German word for determination is *Bestimmung*, build by adding the prefix “Be” to the word *Stimmung*. As we will later see by reference to Schiller, the omission of the determining prefix is taken to indicate the lack of determination in the sense of determinability. Third, the *Stimmung* metaphor implies the process or act of attuning. Most interestingly, this might also be understood in terms of a self-activating attunement (*Einstimmung*) or determination (*Bestimmung*). In this case, the subject and object of attuning are the same and we can speak of a self-attunement (*Selbststimmung*) or self-determination (*Selbstbestimmung*).

## Kant’s Introduction of the *Stimmung* Metaphor

Kant’s *Critique of Judgment* (2007 [1790]) serves as a major turning point in the conceptual history of *Stimmung*. Kant uses the word in order to solve the central problem of his account of beauty. As an ancillary effect, he established *Stimmung* as an aesthetic concept.

The most decisive but also controversial aspect of Kant’s theory of aesthetic judgment – he calls it also judgment of taste or judgment of the beautiful – is the claim that such judgments are subjective but nevertheless universal. Judgments of beauty are subjective in that they are based on feelings, not on concepts. Thus, they cannot have the same objective validity as cognitive judgments. Nevertheless, Kant suggests that a claim to universal validity is a structural feature of such judgments: The judgment that something is beautiful necessarily involves the belief that everyone else who perceives the object ought to also judge it as beautiful. According to the constitution of aesthetic judgments, however, this universality cannot be based on concepts. Instead, Kant explains in § 9 of the *Third Critique* that aesthetic judgment depends on the “free play” or “harmony” of the faculties of imagination and understanding. And he elaborates that this harmony of the faculties is determined by a feeling, not a concept. The crucial step in his argument is the claim that this feeling cannot proceed its communicability. If it were to proceed its communicability, it would be a solely private feeling, which might still be communicated subsequently, but which could not involve the belief that others ought to have it as well. But since a judgment of taste necessarily involves the demand that others ought to judge an object the same way, the order must be the other way around. Thus, Kant concludes that universal communicability is the condition of possibility of the feeling forming the basis of aesthetic judgment.

Now, the key question of Kant’s theory of aesthetic judgment is this: How is it possible that a feeling has universal communicability as its structural feature? To answer this question, Kant resorts to the term *Stimmung*. Kant explains that the harmony of the faculties means that the faculties of imagination and understanding are in the “proportionate *Stimmung*”.^10^ Thus, do describe what it means that the faculties are in harmony with each other, without being forced into harmony by a concept, Kant uses the metaphor of musical attunement. In § 21, he calls the “attunement of the faculties” (*Stimmung der Erkenntniskräfte*) the “state of mind” (*Gemüthszustand*). For Kant, *Gemüt* signifies the principle that unifies the various faculties.^11^ Importantly for our context, Kant thinks of this unification of the mind in terms of its attunement. In short, the universal validity of aesthetic judgments depends on the universal communicability of the state of mind (*Gemütszustand*) which is defined as the attunement of the faculties (*Stimmung der Erkenntniskräfte*).

With this move, Kant established *Stimmung* as an aesthetic concept. It is important to note, however, that Kant’s way of using the term is fully committed to its metaphorical force. His argument depends on the key aspects of musical attunement in such a way that it is impossible to render it in non-metaphorical language. It is the absolute metaphor (Blumenberg 1997) of *Stimmung* that holds the argument together and generates its persuasiveness.

In the previous section, we identified three key features of the *Stimmung* metaphor. We can now see how Kant appropriated all three features for his theory of aesthetic judgment. First, *Stimmung* means the state of being attuned in terms of proportionality or harmony. It is obvious that this is the key aspect that Kant adopts. It allows him to make plausible how the faculties can be in harmony with each other without being unified under a concept. Second, the state of being attuned is understood as a dispositional state. In Kant’s appropriation, this signifies the disposition of the imagination to operate in attunement with understanding. Third, *Stimmung* refers to a process or task, namely the process or task of attuning. According to Kant’s aesthetic theory, it is the self-attuning of the mind that ensures the proportionality of imagination and understanding. Finally, it is also important to note what is missing in this original use of *Stimmung*, namely any reference to an individual’s subjective experience. At the end of the eighteenth century *Stimmung* denotes a dispositional proportionality which is not confined to a subject and her experience, but rather enables universal communicability.

## *Stimmung* as a Lively Metaphor

Kant’s seminal theory of aesthetic judgment was widely discussed among his contemporaries. For the purpose of this paper, I restrict myself to a brief discussion of Friedrich Schiller’s (1982/1962 [1793]) *On the Aesthetic Education of Man*. The aesthetic disposition (*Stimmung*) of the mind plays an essential role in Schiller’s ideas about the education of humans to freedom. The decisive passage is at the end of the 20th letter.

Our psyche [*Gemüt*], then, passes from sensation to thought *via* a middle disposition [*Stimmung*] in which sense [*Sinnlichkeit*] and reason [*Vernunft*] are both active *at the same time*. Precisely for this reason, however, they cancel each other out as determining forces, and bring about a negation by means of an opposition. This middle disposition [*Stimmung*], in which the psyche is subject neither to physical nor to moral constraint, and yet is active in both these ways, pre-eminently deserves to be called a free disposition [*Stimmung*]; and if we are to call the condition of sensuous determination the physical, and the condition of rational determination the logical or moral, then we must call this condition of real and active determinability the *aesthetic*. (Schiller 1982, 141)

In short, Schiller uses the term *Stimmung* to signify a disposition (*Stimmung*) of the mind (*Gemüt*) in which it is neither determined by the senses (*Sinnlichkeit*) nor by reason (*Vernunft*). This clearly draws on Kant, but it also modifies Kant’s idea, as *Stimmung* refers here not only to the proportionality of imagination and understanding, but to a global disposition of the mind – the “aesthetic freedom of determination” which is the disposition of “aesthetic determinability” (Schiller 1982, 145).

According to Schiller, aesthetic determinability implies that the mind is not restricted “by the one-sided constraint of nature in the field of sensation and by the exclusive authority of reason in the realm of thought.” (Schiller 1982, 147) He considers it the power of genuine beauty to induce such a *Stimmung* or disposition in us.

If, by contrast, we have surrendered to the enjoyment of genuine beauty, we are at such a moment master in equal degree of our passive and of our active powers, and we shall with equal ease turn to seriousness or to play, to repose or to movement, to compliance or to resistance, to the discursions of the abstract thought or to the direct contemplation of phenomena.

This lofty equanimity and freedom of spirit, combined with power and vigour, is the mood^12^ [*Stimmung*] in which a genuine work of art should release us, and there is no more certain touchstone of true aesthetic excellence. (Schiller 1982, 151)

Schiller’s use moves the term further away from its musical origin, as proportionality or harmony no longer serves as its core semantic content. But the original metaphor is still very much alive, as it drives the understanding of *Stimmung* as a disposition or ability.

A similar use of the word *Stimmung* as describing a global disposition of the mind can be found in Arthur Schopenhauer’s (1972 [1819]) *The World as Will and Representation*. Schopenhauer conceives of the aesthetic mode of contemplation in terms of two constitutive elements: On the one hand, knowledge of the object in terms of a Platonic idea, i.e. the general form of types of things; on the other hand, self-consciousness of the knowing individual in terms of a “pure will-less subject of knowledge” (Schopenhauer 1972, 231). Thus, Schopenhauer understands the aesthetic state of mind as a state of pure contemplation, in which all individuality is forgotten and knowledge is no longer bound to the principle of sufficient reason. Reaching such an aesthetic state of mind requires the suspension of the will, which allows to contemplate things free from their relation to the will. Such suspension of the will can either happen by an external cause or by an inner disposition (*Stimmung*). Schopenhauer considers it the particular power of such an inner disposition to induce an aesthetic state of mind no matter what the external conditions are. Thus, the focus is here on the aspect of a self-activating attunement (*Einstimmung*) or self-determination (*Selbstbestimmung*).

Furthermore, Schopenhauer explains that the ability to reach such will-less contemplation is the sign of an artistic mind (*künstlerisches Gemüt*), and he takes it to be the artist’s task to display such a state of mind in his work. Accordingly, Schopenhauer deems it a sign of true art that it urges the spectator to participate in the mode of aesthetic contemplation (Schopenhauer 1972, 232). Schopenhauer, thus, follows Schiller’s path of transforming Kant’s connection between the judgment of beauty and the *attunement* of the mind into the connection between the experience of beauty and a *global disposition* of the mind.

Another important step in the conceptual history of *Stimmung* is Carl Gustav Carus’s (2002/1831) *Nine Letters on Landcape Painting*, written between 1815 and 1824. These letters display the expansion of the semantic field of *Stimmung* to include phenomena like landscapes and the weather. Carus is interested in the correspondence between the attunement of the mind and the dynamics of nature. He takes his point of departure in theory of art, claiming that landscape painting has the following principle task: “The representation of a certain mood of mental life [*Stimmung des Gemüthlebens*] (meaning) through reproduction of a corresponding mood of natural life [*Stimmung des Naturlebens*] (truth).” (Carus 2002, 91) Following this definition, he adds an enclosure: “On the Correspondence between Mental Moods [*Gemüthsstimmungen*] and Natural States [*Naturzuständen*]” (Carus 2002, 92–94), in which he is interested in “the specific moods [*Stimmungen*] expressed in the countless metamorphoses of natural landscape.” (Carus 2002, 93) What are these *Stimmungen* of natural life according to Carus? He responds that “all those metamorphoses are simply forms of natural life; therefore, the diverse moods [*Stimmungen*] expressed in them can be nothing other than states of life, stages in the life of nature.” (Carus 2002, 93) In short, Carus takes *Stimmungen* in nature to be nothing but the four phases of life, “evolution, maturity, decline, and extinction”, as they are exemplified in the four seasons or in the four phases of a day, “morning, noon, evening or night.” (Carus 2002, 93) Carus claims that in the same way in which nature is structured by ascending and descending *Stimmungen*, so are the *Stimmungen* of the mind divided into *Stimmungen* of growth and *Stimmungen* of decay. In this sense, the attunements of the mind (*Gemütsstimmungen*) and the attunements of nature (*Naturstimmungen*) correspond to each other.

In contrast to a psychological understanding of *Stimmung*, which will be discussed in the following section, it is important to note that for Carus, these reflections do not beg the question of how it is possible to speak of the *Stimmungen* of nature in any other than a metaphorical sense. For him, there is nothing ontologically problematic about attributing *Stimmungen* to nature in the same literal sense as *Stimmungen* are attributed to the mind. For him, the only issue in need of an explanation is how to conceive of the correspondence between the respective *Stimmungen* in these two domains. He responds to this question by reference to the stages of life, which he takes to be at work in the same way in the life of nature and in the life of the mind.

To end this section, let me briefly mention two prominent figures in nineteenth century thought who are standing in the twilight between the original metaphor and the psychological canonization of *Stimmung*. The first is Friedrich Nietzsche, who, as a student, wrote a short piece entitled “Über Stimmungen” (Nietzsche 1994). In this text, *Stimmung* signifies a complex of feelings whose metamorphoses provide insight into the inner dynamics of psychic life. Similar to Carus, Nietzsche uses *Stimmung* to refer to underlying dynamics of life. But he adds an element of subjectivity to this notion by restricting its usage to the domain of the mind and linking it to felt experience.

This understanding of *Stimmung* as referring to underlying dynamics of life came to prominence in the work of Wilhelm Dilthey. For Dilthey, *Stimmung* denotes the *principium individuationis* of human subjects as well as social collectives. Both in the individual and the collective case, *Stimmung* signifies what can be roughly characterized as an overall character that constitutes a particular perspective on the world. Dilthey developed this thought in the context of *Lebensphilosophie*. Accordingly, he takes *Stimmung* to be more or less synonymous with vital feeling (*Lebensgefühl*). In *Introduction to the Human Sciences* (1883), Dilthey claims that profound cultural change depends on the modification of “vital feelings” (Dilthey 1962, 143 and 355–56). He develops this claim further in *Weltanschauungslehre* (1911), where he discusses life as the source of all world views. The central thought is that “universal moods” or “vital moods” form the “bottom layer for the formation of world views” (Dilthey 1960, 82). It is likely that this formulation was inspired by Henri Bergson (1911), who suggests that, instead of causal or teleological explanations of evolution, we should suppose a primordial “*élan vital*” underlying all life. Dilthey follows this idea of a vital force as the subsoil of life. Based on this premise, he takes *Stimmungen* or vital feelings, deriving from this vital force, as the countless nuances which determine each individual perspective on the world.

We will meet the semantic resources displayed in the works of Kant, Schiller, Schopenhauer, and Carus again at the turn of the twentieth century when Alois Riegl and Georg Simmel reflect on the *Stimmung* of art and landscape. All these thinkers make productive use of the *Stimmung* metaphor, understood as a global attunement or disposition which can be applied equally to nature and the mind. This stands in contrast to the psychological concept of *Stimmung*, which moved the sematic content of the word away from the musical metaphor and reduced *Stimmung* to a subjective experience.

## Psychologizing *Stimmung*

At the turn to the nineteenth century, aesthetics was considered a part of metaphysics, as exemplified by Kant. One century later, aesthetics was firmly established as a sub-field of applied psychology.^13^ The result was the dominance of psychological explanation models of aesthetic experience. Johannes Volkelt’s (1905) *System der Ästhetik* – the major work of this period together with Theodor Lipps’s (1903) *Ästhetik* – paradigmatically displays the dominant understanding of aesthetics as a sub-discipline of psychology.^14^ Volkelt is convinced that form and content of the aesthetic object must be conceived of in terms of human consciousness. In short, the aesthetic object is a psychological entity that needs to be studied with psychological methods.

The psychologizing of aesthetic experience also affected the use of the term *Stimmung*. From Kant’s original transfer of *Stimmung* into the domain of aesthetic theory, psychological aesthetics only adopted the aspect of subjectivity, which it no longer understood in transcendental terms, but in terms of subjective experience. With this transformation, all reference to universal communicability disappeared from its conceptual horizon. Hermann Lotze (1868, 65), for instance, praises Kant for emphasizing the subjective character of aesthetic judgment, but adds that Kant did not go far enough in this direction and that the subjective character needs to be spelled out in terms of a psychological investigation of experience. In the same spirit, it was now seen as the task of psychology to explicate *Stimmung*. This move established the understanding of *Stimmung* as a psychological category.

Lipps and Volkelt were also the founders of the theory of empathy (*Einfühlungstheorie*).^15^ The theory of empathy is the prime example of how aesthetic experience became psychologized. *Einfühlung*, later translated by the neologism *empathy*, was originally introduced as a technical term to signify a specific type of aesthetic experience. It denoted the mental act of *projecting* one’s own self into an object and thereby *vitalizing* or *animating* it. Today, empathy mostly refers to the ability of being sensitive to the thoughts and feelings of others. Initially, however, the understanding of other human beings was only a special case of empathy; the case in which one’s own self was projected into another individual in order to understand him or her. The initial purpose of introducing the term empathy was not for explaining the experience of other living beings, but the explanation of aesthetic experience. Accordingly, Lipps (1909, 223–31) distinguishes four types of empathy: empathy of activity (*Tätigkeitseinfühlung*); empathy of mood (*Stimmungseinfühlung*); empathy into nature (*Einfühlung in die Natur*); and only lastly, empathy into the sensual appearance of other human beings (*Einfühlung in die sinnliche Erscheinung eines Menschen*).

The first decade of the twentieth century saw a number of competing theories for explicating the precise mechanisms of empathy.^16^ From today’s perspective, however, it is more striking to note the common ground in that debate. To begin with, the main premise was that one’s own mind is immediately experienceable, whereas in the case of others, only their bodies are accessible in immediate experience. Accordingly, the minds of others are taken to be experienceable only through the mediation of previous experiences of one’s own mind and the mediation of others’ bodies. Thus, the experience of others is a mediated experience: It moves from my own experience via the experience of other bodies to the experience of other minds.

Much more could be said about this. For the purpose of this paper, these brief remarks serve as a sufficient background for examining the consequences of the psychological framework for the use of the term *Stimmung*. Let me start with Lipps’s notion of empathy into nature (*Einfühlung in die Natur*). He explicates this form of empathy as the case of experiences rhythmizing my soul. When this happens, it leads me to *emphasize* – i.e. project – moods into an object or a situation, which explains how it is possible to experience, for example, a painting as cheerful or a landscape as frightening. Lipps’s theory shows that the musical metaphor is still at play even in the psychological understanding of *Stimmung*. However, *Stimmung* is now fully transformed into a psychological concept. Within a psychological framework, it is obvious that all *Stimmungen* or moods are located in individual minds. It is psychological common sense that only sentient beings can have feelings, emotions or moods. Thus, if moods are attributed to objects, there is no other way of understanding this than in terms of a projection (Prandtl 1910, 89–106). And this is also the precise meaning of the theory of empathy: *Einfühlung* literally means *to feel something into something else*. Hence, speaking of empathy implies the idea that, for example, when someone experience a meeting as tense, this needs to be explained psychologically in terms of her projecting her own mental states into the meeting (Lipps 1909, 226).

In brief, within psychological discourse, there is only one possible way of understanding *Stimmung*, namely as a state of psychic arousal. Only subsequently, *Stimmung* might be projected into objects. The object itself, however, can never be in *Stimmung*. Within a psychological framework, objects, may they be natural objects or artifacts like artworks, simply do not qualify as bearers of *Stimmung*. *Stimmung* is exclusively understood as an attribute of the mind, an attunement of the soul, which might be caused by the experience of objects, but which can never be attributed to objects, except in terms of a projection (Witasek 1904, 99).

Let me end this section with a discussion of the most elaborate work on *Stimmung* in the context of psychological discourse at the beginning of the twentieth century. In 1911, the German philosopher Moritz Geiger, a disciple of Lipps and member of the Munich phenomenological circle, published a text on the problem of empathy of mood (*Stimmungseinfühlung*) (Geiger 1911b). Whereas the empathy of *Stimmung* was only a secondary theme in the debate on empathy, Geiger sets out to clarify what we main when speaking of a lovely landscape or a buoyant color. Geiger (1911b, 4) follows Lipps in distinguishing between empathy of activity (*Tätigkeitseinfühlung*) and empathy of mood (*Stimmungseinfühlung*). For the latter, he identifies two explanatory theories. The theory of animation (*Belebungstheorie*), which claims that objects actually possess mental states, and the theory of effect (*Wirkungstheorie*), which holds that the mood ascribed to an object is in fact the feeling a subject has based of the effect which the object has on her. As Geiger notes, all psychologists of his time supported the latter theory.

Geiger develops a complex argument in favor of a third alternative. Against the theory of effect, he emphasizes the phenomenological fact that we are able to register mood-like qualities of objects like a landscape without having to presuppose either an equivalent mood in the subject or an animation of the object. About two decades later, Heidegger appears to build on Geiger’s claim in his lecture course *The Fundamental Concepts of Metaphysics* (1929/30). Let us briefly consider Heidegger’s (1995, 82–88) argument here, as it contributes to the force of Geiger’s case. If the boredom of a book stood in a cause-effect relation with my mood, as the theory of effect maintains, the boredom of the book would always cause me to be in a bored mood, just as a thermometer always shows a lower figure when the temperature falls. However, this is not the case: A boring book can lead to a variety of affective responses; one might be bored by a boring book, but one might also be upset by it. Thus, it is implausible that the boredom of a book and my mood stand in causal relation. Moreover, if the attribution of boredom to the book were only a projection of my mood, it would be incomprehensible how different objects can be perceived differently while being in the same mood. However, even when someone is in a particular mood, she is capable of experiencing different things in different ways. For instance, when someone is irritated, this does not deprive her from the ability to experience one individual as friendly and another one as rude. As a consequence, the *Stimmungen* we perceive in objects cannot simply be projections based on the effects which objects have on our minds.

Geiger (1911b, 9) paths the way towards an alternative explanation by introducing a distinction between *Stimmung* in terms of the *feeling* of a subject and *Stimmung* in terms of the *character* of an object. As shown by Heidegger, the major flaw of the theory of effect becomes particularly obvious when considering cases where the subject’s feeling stands in tension with the object’s character. For instance, the gloomy character of an artwork might be particularly conspicuous when encountering it in a lifted mood. Thus, the character of an object cannot be reduced to the effect it has on the mind of a subject, since the relation of the two is not one of simple causality, as the theory of effect would have it.

Geiger aims at illuminating these intricate matters with help of the phenomenological distinction between noetic and noematic content. This allows him to introduce a further differentiation within a subject’s mood. On the noetic side, he identifies the mental act-process of a mood, which is a subjective feeling. On the noematic side, there is the internal object of the act, which he calls the “feeling tone” (*Gefühlston*) (Geiger 1911b, 17). The feeling tone can impose itself upon external objects, making them appear cheerful when in a lifted mood and gloomy when in a bad mood. Now, the decisive question in Geiger’s solution concerns the relationship between the *feeling tone*, as part of a subject’s mood, and the *character* of an object. Geiger’s submits that there is a qualitative kinship between the two. When this kinship is realized in experience, he suggests speaking of the “feeling character” (*Gefühlscharakter*) of an object (Geiger 1911b, 20). Furthermore, he suggests that there is a continuous interplay between the feeling character of objects and the mood of a subject, and that this interplay is precisely what happens in the experience of, for instance, a landscape (Geiger 1911b, 21).

In my estimation, Geiger’s solution is the closest one can get to the attribution of *Stimmung* to objects within a psychological framework, and it would certainly be worth further exploration and debate.

## The Ongoing Potential of the Metaphor

Parallel to psychological discourse, there continued to be approaches that aligned more closely to the way the *Stimmung* metaphor was used around 1800. More specifically, a conceptual lineage can be drawn from Schiller, Schopenhauer and Carus to the Austrian art historian and member of the Vienna School of Art History Alois Riegl.

In a short text from 1899, “Die Stimmung als Inhalt der modernen Kunst” (*The Stimmung as the content of modern art*), Riegl (1929) asks about the organizing principle in each epoch of mankind, suggesting that humanity’s need of harmony was met differently in each epoch. In early times, characterized as a war of all against all, it found its expression in fetischism. Antiquity saw it in the law of the strongest. Suggesting that the function of art corresponds with the dominant ordering principle, Riegl sees this as the reason why Ancient art celebrated physical strength. The emergence of Christianity signified the wish for a moral world order; thus, Christian art had the task of glorifying the moral superiority of God, which meant that spiritual integrity instead of physical strength became its main theme. Finally, the modern scientific world view expected the explanation of natural phenomena in terms of knowledge, not faith. Riegl claims that knowledge can provide the relieving harmony if we observe nature from a distant view. This is the task of modern art. Just as the scientific world view understands all occurrences as part of the causal nexus of nature, modern art also places them in such an order. It is the peculiarity of Riegl’s understanding of modernity that he considers its longing of harmony fulfilled in the sentimental awareness of the cosmological order of natural laws (Riegl 1929, 30–33).

Intriguingly for our purpose, Riegl calls this awareness *Stimmung*. In short, *Stimmung* signifies the calming awareness that nature abides by the laws of causality. This makes Riegl a predecessor of Leo Spitzer’s (1963, 1) claim that it is “the concept of world harmony which underlies the word Stimmung.” In the context of the conceptual history presented so far, two aspects of Riegl’s notion of *Stimmung* are particularly striking. First, Riegl follows a Kantian use of *Stimmung* by understanding it as a communicable state of mind. However, he does not argue for its universality. Rather, he adopts a historistic perspective, claiming that *Stimmung* is a particularly modern phenomenon. Second, Riegl’s notion of *Stimmung* is, on the one hand, very general; on the other hand, it is obtained with reference to a specific experiential domain. Riegl introduces *Stimmung* with help of the image of a hiker who oversees the surrounding area from a mountain height: The removed position allows to see peaceful harmony where direct involvement exhibits relentless fight. In brief, *Stimmung* signifies the sentiment of harmony that overcomes chaos, dissonance and movement. Achieving *Stimmung* requires rest and a distant view (*Ruhe und Fernsicht*), while every urge to move makes it disappear (Riegl 1929, 27–28).

Whereas nature only grants *Stimmung* in rare moments, Riegl (1929, 29) sees it as the task of modern art to provide this comforting awareness of order and harmony. This shows Riegl in line with Schopenhauer (1972, 232), who suggested that the particular power of an artistic mind (*künstlerisches Gemüt*) lies in its ability to represent an aesthetic state of mind (*ästhetischen Gemütszustand*) and to urge the spectator to participate in such a state of mind. Furthermore, Riegl (1929, 33) suggests that the visual arts are most suitable for this task, for only visual forms – and the calm and distant view with which they correspond – have the power to communicate the harmonious workings of the law of causality. With this claim, he again follows in the footsteps of Schopenhauer who suggested that still lives and landscape paintings are particularly suitable for the task of eliciting an aesthetic state of mind, as they display the calmness and contemplation necessary for attentive observation (Schopenhauer 1972, 232).^17^

This focus on landscapes, which can be traced back to Carus (1831), was picked up by Georg Simmel, one of the founding fathers of German sociology. In the short text “Philosophy of landscape” (1913) he seeks to examine *landscape* as an object of art – exemplified in landscape painting – and as an object of everyday experience (Simmel 2007/2001). Simmel follows Riegl’s historistic approach, claiming that landscape is an exclusively modern phenomenon. Experiencing a landscape means to demarcate a particular formation within the entirety of nature. Simmel suggests that it was modernity’s process of individualization which made such a demarcation possible. By contrast, Antiquity and the Middle Ages had no awareness of such distinct formations within nature, as they related to nature as a totality which did not lend itself to being divided into discrete parts.

Simmel explains that “a landscape arises when a range of natural phenomena spread over the surface of the earth is comprehended by a particular kind of unity” (Simmel 2007, 26). This leads to the central question in Simmel’s text: What constitutes this particular kind of unity defining a landscape? Simmel’s answer is: *Stimmung*. This is the crucial passage:

When we refer to the mood [*Stimmung*] of a person, we mean that coherent ensemble that either permanently or temporarily colours the entirety of his or her psychic constituents. It is not itself something discrete, and often also not an attribute of any one individual trait. All the same, it is that commonality where all these individual traits interconnect. In the same way, the mood [*Stimmung*] of a landscape permeates all its separate components, frequently without it being attributable to any one of them. In a way that is difficult to specify, each component partakes in it, but a mood [*Stimmung*] prevails which is neither external to these constituents, nor is it composed of them. (Simmel 2007, 26)

On the one hand, the first half of the citation shows Simmel’s reference to psychological discourse, understanding the mood of a person in terms of subjective experience. On the other hand, *Stimmung*, for Simmel, has the task of being a unifying principle, similar to how the metaphor was originally introduced. It signifies the unity of a whole which is more than its parts. This appears to be the core semantic content of Simmel’s use of the term *Stimmung*, and he takes this semantic content to be applicable to individual minds as well as to quasi-objects like a landscape.

Now, this leads to a crucial ontological question: Who or what is the carrier of the unity of a landscape? Like Geiger, Simmel considers both, the theory of animation and the theory of effect, as wanting. But he is more radical than Geiger in transcending the psychological framework. His goal is an encompassing anthropological perspective that does not require one to reduce *Stimmung* to the mental states of individuals. It is clear for Simmel that a solution must overcome the subject-object-dualism. Thus, he claims that “the *Stimmung* of a landscape and the perceptual unity of a landscape are one and the same thing.” (Simmel 2007, 27) A landscape can neither be reduced to a projection of the mind, nor to a number of physical attributes. In other words, quasi-objects like landscapes only become conceivable for a perspective which neither focuses on our mind, nor on the physical makeup of the world, but which considers the primordial relatedness of mind and world. However, Simmel fails to develop this idea into a comprehensive theory that can serve as an alternative to the psychological framework. I submit that this is precisely the aim of Heidegger’s *Being and Time*.

## *Stimmung* as a Resource for an Alternative Ontology

It is fair to say that the discourse on *Stimmung* fundamentally changed with the publication of Heidegger’s (1996/1927) *Being and Time*. This work has the revolutionary potential of providing a powerful ontological alternative to the psychological framework. Knowing the conceptual history of the word *Stimmung* enables one to see that Heidegger built on the resources that the historical lineage from Kant to Simmel provided. At the same time, it also allows to appreciate Heidegger’s unique contribution of condensing those resources into the comprehensive ontological alternative presented in *Being and Time*.

The most salient aspect of earlier usage adopted by Heidegger is the idea that *Stimmung* refers to wholes, or more precisely, to the principle of unification of wholes. For Kant, Schiller and Schopenhauer, *Stimmung* denoted the unifying principle of the mind. In Dilthey, it signified the unity of world views. For Riegl, it was the awareness of the whole of nature abiding to the law of causality. Finally, Simmel identified it as constituting the unity of a landscape. Thus, *Stimmung* serves a unifying function, integrating elements (whether capacities of the mind or parts of nature) into wholes that are more than their parts.

Heidegger built on this idea with his central claim that *Stimmung* attunes being-in-the-world as a whole. To defend this claim, it is crucial for Heidegger to oppose any psychological interpretation of *Stimmung*. He continuously emphasizes that it is a fundamentally flawed approach to discuss *Stimmung* in terms of psychology: *Stimmungen* cannot be conceived in terms of mental states, they are not occurrences within a mind (Heidegger 1995, 63–66). More generally, *Stimmungen* are neither in a subject, nor in an object. Rather, they attune the relationship as a whole. This is the case both in terms of being-with-one-another (intersubjectivity) and in terms of being-amidst entities (subject-object relation). In other words, *Stimmungen* serve a basic function in shaping our relationships with others and with things.

The primacy of being-in-the-world as a relationship is so central to Heidegger’s thought that it serves as the basis for the layout of the first division of *Being and Time*. The first division of the book analyses being-in-the-world as a unified structure, only analytically focusing on the world (chapter 3), the self (chapter 4), and on being-in as such (chapter 5). *Befindlichkeit*, defined as the ontological condition of always already being in a *Stimmung*, is introduced in chapter 5 as one of the three existentiale of being-in (besides understanding and discourse) (Heidegger 1996, 126–31). Thus, *Stimmung* comes into view when considering being-in-the-world in terms of a relational totality. More precisely, *Stimmung* is what attunes the relation itself and, thus, is constitutive of being-in-the-world as a whole.

In his attempt to overcome established dualisms, Heidegger avoids speaking of subject and object, just as he avoids speaking of mind and body. Thus, it is difficult – and in opposition to the spirit of his work – to translate Heidegger’s thought back into subject-object terminology. If one nevertheless wants to do so, one could say that *Stimmung* needs to be conceived as subject-related and object-related at the same time, as it is located in the dynamic relation of a subject to its world, or more appropriately, as it attunes this relation as a whole. In a terminology that is closer to *Lebensphilosophie*, one might say that living in the sense of being-in-the-world means to live in a meaningful world which one encounters as relevant or mattering. Being affected by things mattering to me is what Heidegger calls the *Stimmung* of being-in-the-world. In sum, *Stimmung* is a key concept of Heidegger’s alternative to the psychological framework.^18^

## Epilogue

After this brief discussion of Heidegger‘s *Being and Time* as the second major turning point in the conceptual history of the term *Stimmung*, this paper ends with an outlook into the developments that have taken place after Heidegger, followed by some tentative implications for current theorizing on mood and atmosphere.

Without any claim of being exhaustive, I want to highlight three traditions. First, Heidegger’s notion of *Stimmung* was developed further by some of his disciples, most importantly by Otto Friedrich Bollnow (1953). This line can be traced further via Stephan Strasser (1956) to Matthew Ratcliffe’s (2005, 2008) notion of “existential feelings.” Second, Merleau-Ponty’s (2012 [1945]) phenomenology of *intercorporeality* and *interaffectivity*, which is currently advanced by Thomas Fuchs (2013, 2017) among others, offers another relational and dynamic account of affectivity. Third, Hermann Schmitz’s neo-phenomenology added another perspective by defending an objective understanding of feeling. According to Schmitz (1969), feelings are objective occurrences and need to be distinguished from an individual’s affection by them. Hence, Schmitz conceives of all feelings as atmospheres. Gernot Böhme (2017) developed Schmitz’s idea into an aesthetic theory centered around the notion of atmosphere; an approach that is currently also defended by Tonino Griffero (2014, 2017). All three traditions as well as their relation to each other would be worth further exploration. This obviously lies beyond the scope of this paper.

What can be noted is that the conceptual resources we encountered in the conceptual history of the word *Stimmung* do not play a role in any of these traditions. The core idea of the first two traditions is an understanding of vital feelings as the ground layer of human affectivity. The third tradition, by contrast, is centered around the notion of atmosphere as a trans-subjective determination of situations and spaces. Thus, despite these ongoing traditions, or maybe in light of them, David Wellbery (2003, 733) suggests that the semantic of *Stimmung* has become dull and that the musical metaphor, which made *Stimmung* such a productive notion in nineteenth century thought, has lost its evidence. In this paper, this loss of evidence and the trivialization of the use of *Stimmung* was traced back to the increasing dominance of a psychological framework which leaves no room for the metaphorical complexity that was vivid from Kant to Heidegger.

On the other hand, growing research interest in moods and atmospheres can be interpreted as a sign that the need for alternatives beyond the psychological framework is increasingly felt. At this junction, it seems that all three traditions outlined above could profit from more knowledge about the complex history of the basic terms around which they are build. This would allow for heightened awareness of the available conceptual alternatives and the issues at stake in each of them. In particular, the *Stimmung* metaphor could provide as yet underappreciated conceptual resources. It has been shown in this paper how *Stimmung* serves as a unifying principle of wholes that are more than their parts; a dynamic that is applicable to subjects and objects alike. More accurately, *Stimmung* leads one to focus on relations instead of substantive poles and, thus, has the potential for thinking beyond the subject-object dualism. Moreover, *Stimmung* points to a dispositional state of openness for further determination (*Bestimmung*), both in terms of a self-activating and a foreign-affected attunement. It thus aligns well with a relational and processual ontology (Renault 2016) that emphasizes relationality, process and interactivity over individual states and fixed and stable conditions (Slaby and von Scheve 2019).

It remains to be seen whether Wellbery is right that the *Stimmung* metaphor is dead, or whether we will see a revival of *Stimmung* as an untranslatable, irreducible metaphor with unique semantic force allowing for original theorizing.

## Footnotes

1 The most important work on the history of the word *Stimmung* is (Wellbery 2003). The present paper is much indebted to his pioneering research. The first text on *Stimmung* in a historic perspective was (Spitzer 1963), although with a special thesis centered around the notion of “world harmony.” As the purpose of the present paper is an intervention into current philosophical debate, I focus on the use of the term *Stimmung* in scholarly texts with a particular focus on texts by relevant figures within the history of philosophy. This purpose distinguishes the present paper from Wellbery and Spitzer, who both were philologists and approached the issue from the perspective of literary theory. As I will show in footnote three, literary theory continues to be the dominant discipline in current research on *Stimmung*. A final remark on methodology: Although I use the term conceptual history in the subtitle, I do not bind myself to the methodology of *Begriffsgeschichte* (Koselleck 2002).

2 Regarding current interest in moods, see (Stephan 2017; Ringmar 2017). This new interest in moods is connected to a renewed interest in the work of Martin Heidegger and his possible contribution to the understanding of affectivity (Ratcliffe 2013; Withy 2014, 2015; Slaby 2015, 2017). The current rise of interest in atmospheres is driven by English editions of important works by Gernot Böhme (2017) and Tonino Griffero (2014, 2017), who both build on the work of the German neo-phenomenologist Hermann Schmitz (1969). Moreover, the popularity of affect theory has likely contributed to the renewed interest in atmospheric phenomena (Massumi 1995; Protevi 2009). Inspired by Gilles Deleuze and Félix Guattari, new trajectories of thinking about the social are explored. This has, for instance, led to the rediscovery of Gabriel Tarde and his psychology of the crowd (Tarde 1890; cf. Candea 2010), which has recently been linked with the neo-phenomenological notion of atmosphere (Runkel 2018).

3 Since the publication of Wellbery’s (2003) seminal text, there has been growing research interest in the word *Stimmung*, in particular in literature studies. See for instance the works of Hans Ulrich Gumbrecht (2011), Hans Georg von Arburg (2012), and Caroline Welsh (2006, 2008, 2009a, b). For research on *Stimmung* from the perspective of musicology see (Moosmüller et al. 2017).

4 The most important philosophical dictionary in German, *Historisches Wörterbuch der Philosophie* (Ritter et al. 1971–2007), has only a very short entry on the word *Stimmung*. The main claim of this entry is that the word *Stimmung* only gained a philosophical profile towards the end of the nineteenth century in the context of *Weltanschaungsphilosophie*. As this paper will show, this claim is rather misleading. Despite mentioning Herder, Kant and Schopenhauer, the entry makes the dubious claim that the history of *Stimmung* as a philosophical term first started with Dilthey. This claim is also meant to justify that more than half of the entry is awarded to the discussion of Heidegger and Bollnow. The only other philosophers mentioned are Cassirer and Ryle (Wetz 1998). In the main dictionary in the tradition of *Begriffsgeschichte* (Brunner et al. 2004), there is no entry on *Stimmung*.

5 As will be shown in the next section, the etymology of *Stimmung* differs significantly from that of English terms that might serve as a translation. It is arguable one of most intriguing aspects of the word *Stimmung* that it has no direct equivalent in other major European languages. For that reason, I will use various translations throughout this paper and often leave the term untranslated.

6 Despite this etymology, it has become common among Heidegger scholars to employ the term as translation of *Befindlichkeit*, which is the ontological condition of being in *Stimmung* (Heidegger 1996, 126–31).

7 For this paragraph, see the entry on mood in the Oxford English Dictionary.

8 In nowadays colloquial German *Mut* means courage. However, the original meaning is still present in old names of moods or dispositions like *Schwermut* (melancholia), *Hochmut* (pride), *Sanftmut* (gentleness) or *Demut* (humility).

9 The term *atmosphere* cannot really function as a translation of *Stimmung*, but let me briefly mention it here anyway. It was coined in the seventeenth century as a neologism from the Greek terms *atmos* (vapour) and *sphaira* (sphere), signifying a “gaseous envelope” surrounding an object, for example a planet. For a conceptual history of the word atmosphere see (Riedel 2019).

10 Meredith (Kant 2007) translates *Stimmung* here as “accord”, Pluhar (Kant 1987) chooses “attunement”. I side with Pluhar and use attunement in the following sentences.

11 The term is usually translated in English-speaking Kant scholarship as *mind*. As was mentioned in the previous section, *Gemüt* has the same origin as the English *mood*. It is built by adding the prefix “Ge”, which indicates a collection or gathering. Thus, *Gemüt* is the collection or gathering of the faculties of the mind.

12 *Mood* is a misleading translation here insofar as it implies the element of subjective experience, which is not present in Schiller’s use of the term. Hence, *disposition* seems to be a more suitable translation in this context.

13 See for example the beginning of Theodor Lipps’s (1903, 1) aesthetics, probably the most important work of psychological aesthetics at the turn of the twentieth century. Advocates of the psychological movement appealed to romanticism as precursors for their understanding of aesthetic experience. This connection, however, is probably less pronounced than they make it appear (Stern 1898, 1–8).

14 Despite their relevance at the time, Lipps and Volkelt are mostly forgotten today. In the case of Lipps, it is also noteworthy that he played a crucial role for the formation of the early phenomenological movement. His disciples – among them Moritz Geiger, Alexander Pfänder, and Adolf Reinach – formed the so-called Munich circle of phenomenology. For an overview of the Munich Circle see (Spiegelberg 1960, 171–218).

15 For a detailed discussion of the initial theory of empathy see (Schloßberger 2005).

16 Besides the works of Lipps and Volkelt see also (Stern 1898; Witasek 1904; Prandtl 1910). For an overview of the debate see (Geiger 1911a).

17 More specifically, Riegl (1929, 34) suggests *Stimmung* is best achieved when mobility is portrayed without the depiction of actual movement. He thus thinks that *Stimmung* is particularly appropriate for the representation of botanic and inanimate nature, insofar as its movement follows natural laws. Riegl takes this to explain why landscape painting took the most distinguished place in modern arts.

18 For detailed discussions of Heidegger’s notions of *Befindlichkeit* and *Stimmung* see (Ratcliffe 2013; Slaby 2015; Thonhauser 2020)
